# Supplementary material for: Enzymatic synthesis of lignin derivable pyridine based polyesters for the substitution of petroleum derived plastics
Source: Nat Commun. 2019 Apr 16;10:1762. doi: 10.1038/s41467-019-09817-3 (PMC6467960; doi:10.1038/s41467-019-09817-3)
Supplement: Supplementary file 1 — Supplementary Information [file 41467_2019_9817_MOESM1_ESM.pdf]

Supplementary Information

Enzymatic synthesis of lignin derivable pyridine based polyesters for the substitution of petroleum derived plastics

Alessandro Pellis[1, *], James W. Comerford[1], Simone Weinberger[2], Georg M. Guebitz[2,3], James H. Clark[1] and Thomas J. Farmer[1,*]

[1] The University of York, Department of Chemistry, Green Chemistry Centre of Excellence, YO10 5DD, Heslington, York, UK
[2] University of Natural Resources and Life Sciences Vienna, Department of Agrobiotechnology, Institute of Environmental Biotechnology, Konrad Lorenz Strasse 20, 3430, Tulln an der Donau, Austria
[3] Austrian Centre of Industrial Biotechnology, Division Enzymes & Polymers, Konrad Lorenz Strasse 20, 3430, Tulln an der Donau, Austria
* Correspondence to: Dr. Alessandro Pellis, ale.pellis@york.ac.uk, alessandro.pellis@gmail.com and Dr. Thomas J. Farmer thomas.farmer@york.ac.uk

Supplementary Tables

Supplementary Table 1. Determination of the monomer's melting points via DSC analysis.

Diester	Melting temperature [ºC]
Diethyl pyridine-2,4-dicarboxylate (PD24)	28
Diethyl pyridine-2,5-dicarboxylate (PD25)	46
Diethyl pyridine-2,6-dicarboxylate (PD26)	44
Diethyl isophthalate (DEI)	Liquid at RT*
Diethyl terephthalate (DET)	44
Diethyl-2,5-furandicarboxylate (DEF)	45
Dimethyl pyridine-2,4-dicarboxylate	Not evaluated
Dimethyl pyridine-2,5-dicarboxylate	216
Dimethyl pyridine-2,6-dicarboxylate	124
Dimethyl isophthalate	68
Dimethyl terephthalate	144
Dimethyl-2,5-furandicarboxylate	110

Supplementary Table 2. iCaLB-catalyzed synthesis of pyridine diesters-based polyesters in a solventless reaction system at 70 or 85 ºC

Diester	Diol	T [ºC]	P [mbar]	Conv. [%][a]	M_n [Da][b]	M_w [Da][b]	Đ[b]
PD24	BDO	70	1000/20	72	500	700	1.3
PD25				47	400	500	1.0
PD24		85		82	2100	4400	2.1
PD25				78	1200	1900	1.6

[a] Calculated via ^{1}H-NMR based on the used diester monomer
[b] Calculated via GPC using a polystyrene calibration curve

Supplementary Table 3. iCaLB-catalyzed synthesis of pyridine diesters-based polyesters in a solventless reaction system at 85 ºC and 20 mbar.

Diester	Diol	Conv. [%][a]	M_n [Da][b]	M_w [Da][b]	D[b]
PD24	BDO	89	800	1400	1.7
	HDO	88	1300	2900	2.2
	ODO	89	1800	4200	2.3
PD25	BDO	86	600	800	1.4
	HDO	88	900	1300	1.6
	ODO	83	1000	1800	1.8
PD26	BDO	86	600	700	1.2
	HDO	88	800	1100	1.5
	ODO	88	1300	2200	1.7

[a] Calculated via ^{1}H-NMR based on the used diester monomer
[b] Calculated via GPC using a polystyrene calibration curve

Supplementary Table 4. iCaLB-catalyzed synthesis of terephthalate, isophthalate and furandicarboxylate-based polyesters in a solventless reaction system at 85 ºC and 20 mbar.

Diester	Diol	Conv. [%][a]	M_n [Da][b]	M_w [Da][b]	D[b]
DET	BDO	89	600	800	1.2
	HDO	89	1100	1800	1.7
	ODO	85	1200	2100	1.8
DEI	BDO	91	900	1500	1.6
	HDO	93	2400	5800	2.5
	ODO	98	2600	7200	2.8
DEF	BDO	81	600	900	1.3
	HDO	94	700	1200	1.6
	ODO	97	800	1500	1.8

[a] Calculated via ^{1}H-NMR based on the used diester monomer
[b] Calculated via GPC using a polystyrene calibration curve

Supplementary Table 5. iCaLB-catalyzed synthesis of pyridine diesters-based polyesters in a DPE reaction system at 85 ºC and 20 mbar.

Diester	Diol	Conv. [%][a]	M_n [Da][b]	M_w [Da][b]	D[b]
PD24	BDO	89	2100	4400	2.1
	HDO	97	5900	17600	3.0
	ODO	99	14300	32100	2.2
PD25	BDO	85	1200	1900	1.6
	HDO	95	4800	10800	2.2
	ODO	98	8100	12100	1.5
PD26	BDO	91	600	700	1.3
	HDO	99	2200	4300	2.0
	ODO	99	3200	7000	2.2

[a] Calculated via ^{1}H-NMR based on the used diester monomer
[b] Calculated via GPC using a polystyrene calibration curve

Supplementary Table 6. iCaLB-catalyzed synthesis of terephthalate, isophthalate and furandicarboxylate-based polyesters in a DPE reaction system at 85 ºC and 20 mbar.

Diester	Diol	Conv. [%][a]	M_n [Da][b]	M_w [Da][b]	D[b]
DET	BDO	95	1300	1600	1.2
	HDO	99	3000	4500	1.5
	ODO	97	6300	9900	1.6
DEI	BDO	96	2400	4100	1.7
	HDO	99	2700	8900	3.3
	ODO	99	3200	15800	5.0
DEF	BDO	94	1300	1900	1.4
	HDO	96	2700	4700	1.8
	ODO	96	3700	5900	1.6

[a] Calculated via ^{1}H-NMR based on the used diester monomer
[b] Calculated via GPC using a polystyrene calibration curve

Supplementary Table 7. DSC analysis of the polymers synthesized from DEI and PD24 in the bulk reaction system.

Diester	Diol	Tg [°C]			ΔCp J/(g °C)
PD24	BDO	-19	-14	-10	0.51
	HDO	-29	-25	-20	0.50
	ODO	-29	-25	-22	0.55
DEI	BDO	-18	-15	-12	0.45
	HDO	-18	-15	-13	0.45
	ODO	-28	-25	-22	0.48

Supplementary Table 8. DSC analysis of the polymers synthesized from DET, DEF, PD25 and PD26 in the DPE reaction system.

Diester	Diol	T_c [°C]	First T_m [°C]	Second T_m [°C]
PD25	BDO	90	-	105
	HDO	71	91	100
	ODO	78	85	95
PD26	BDO	105	-	133
	HDO	41	87	100
	ODO	28	-	88
DET	BDO	112	122	126
	HDO	100	90	106
	ODO	89	97	103
DEF	BDO	83	-	117
	HDO	84	-	111
	ODO	96	-	118

Supplementary Table 9. DSC analysis of the polymers synthesized from DEI and PD24 in the DPE reaction system.

Diester	Diol	T_g [°C]			ΔCp J/(g °C)
PD24	BDO	23	28	31	0.35
		25	29	32	0.35
	HDO	6	10	13	0.41
		6	9	11	0.42
	ODO	-8	-4	-4	0.40
		-5	-2	-1	0.40
DEI	BDO	5	9	9	0.29
		4	8	9	0.31
	HDO	-4	-1	-0	0.33
	ODO	-27	-24	-23	0.45

Supplementary Table 10. DSC analysis. DSC analysis of the polymers synthesized from DET, DEF, PD25 and PD26 in the DPE reaction system.

Diester	Diol	T_c [°C]	First T_m [°C]	Second T_m [°C]
PD25	BDO	140	-	157
		140	-	155
	HDO	104	122	131
		105	121	130
	ODO	102	118	127
		106	120	128
PD26	BDO	145	-	165
	HDO	89	111	128
	ODO	56	86	100
		48	86	102
DET	BDO	169	-	180
	HDO	122	129	140
	ODO	113	124	132
DEF	BDO	125	140	151
	HDO	116	134	145
	ODO	121	136	144

Supplementary Table 11. TGA analysis of the aromatic-aliphatic polyesters synthesized in DPE.

Ester	Diol	T_d5 [°C]	T_d10 [°C]	T_d50 [°C]
PD24	BDO	306	319	348
		312	324	350
	HDO	325	336	369
		321	336	367
	ODO	324	338	366
		333	343	371
PD25	BDO	288	308	352
		295	311	348
	HDO	334	345	375
		332	344	377
	ODO	336	352	377
		338	348	376
PD26	BDO	289	316	367
	HDO	333	349	384
	ODO	336	351	388
		337	352	389
DET	BDO	360	372	401
	HDO	370	379	401
	ODO	372	380	401
DEI	BDO	344	362	397
	HDO	366	376	399
	ODO	373	382	399
DEF	BDO	311	345	388
	HDO	355	367	394
	ODO	358	367	388
		360	369	387

Supplementary Table 12. MALDI analysis of the aromatic-aliphatic polyesters synthesized in DPE.

Polymer		End groups		
Ester	Diol	Ester/diol	Cyclic	Ester/ester
PD24	ODO	Main	Present	X
PD25	HDO	Main	X	x
PD26	BDO	Main	Present	Present

Supplementary Figures

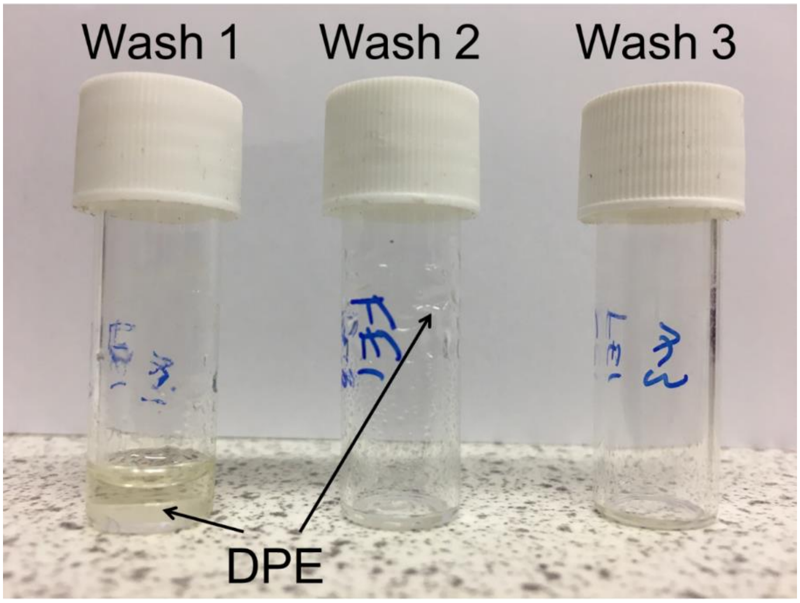

Supplementary Figure 1. Non-volatile component recovered from the different washing steps performed after the enzymatic synthesis of poly(1,4-butylene furanoate). After removal of the volatile component/precipitation agent MeOH under vacuum (24 h, 21 ºC, 35 mbar).

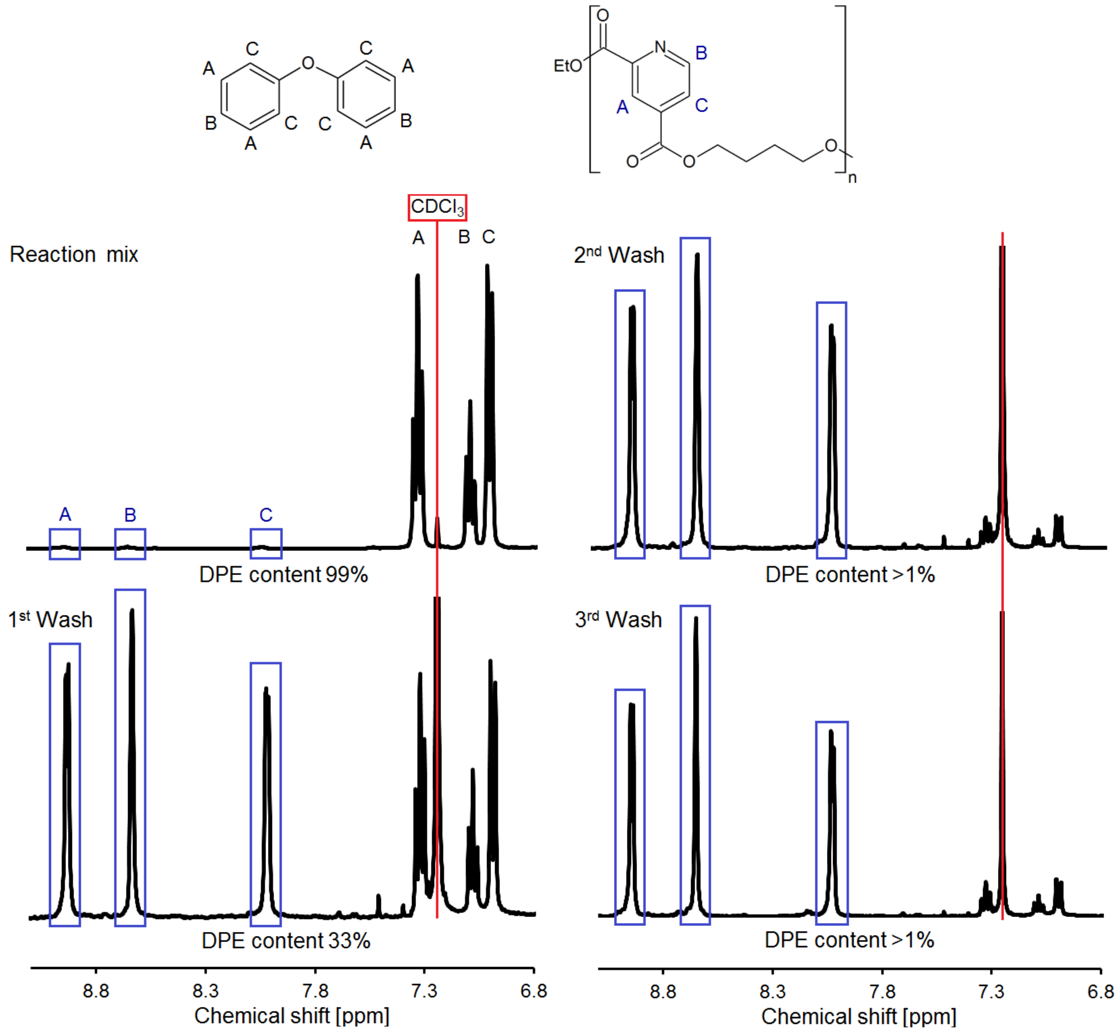

Supplementary Figure 2. ^{1}H-NMR analysis of the poly(1,4-butylene 2,4-pyridinoate) sinthesized in DPE before and after the various purification steps.

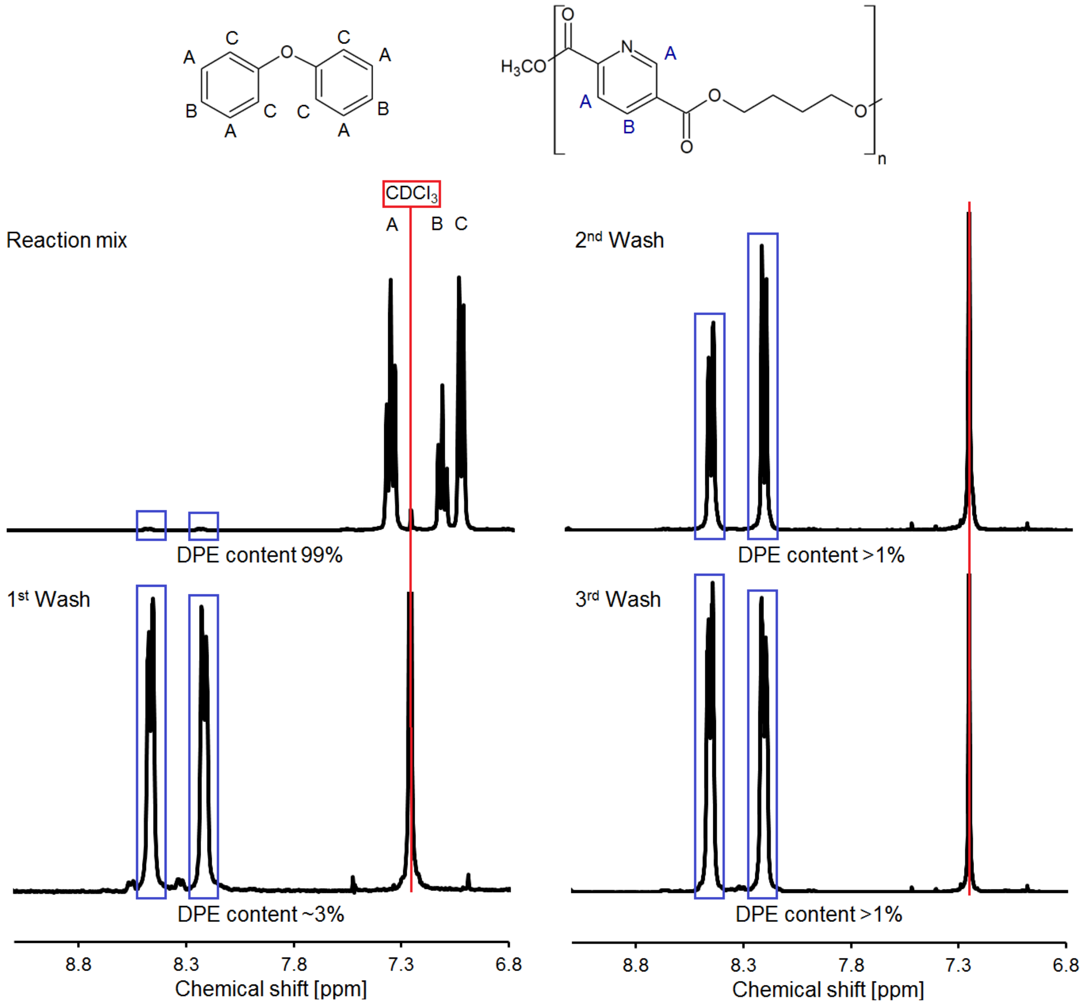

Supplementary Figure 3. ^{1}H-NMR analysis of the poly(1,4-butylene 2,5-pyridinoate) sinthesized in DPE before and after the various purification steps.

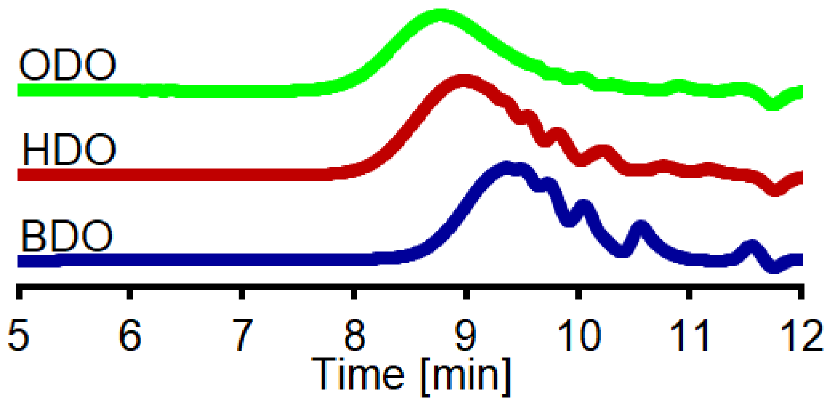

Supplementary Figure 4. GPC chromatograms of the polymers synthesized from diethyl-2,4-pyridinedicarboxylate and the three aliphatic diols.

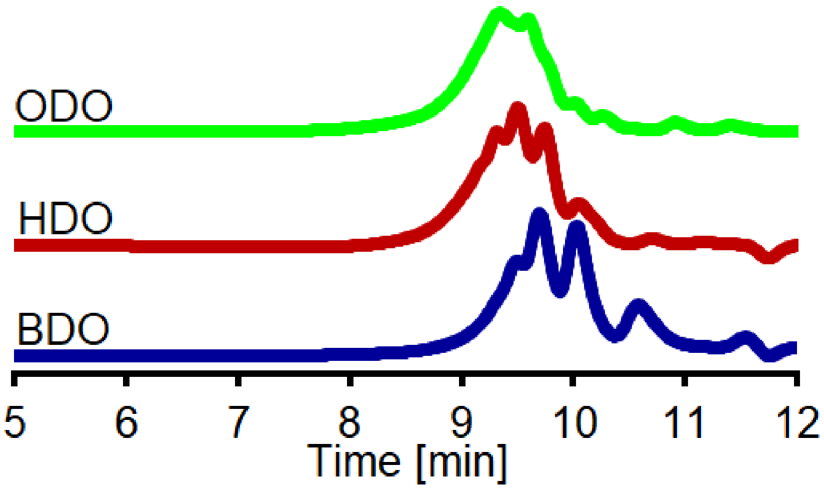

Supplementary Figure 5. GPC chromatograms of the polymers synthesized from diethyl-2,5-pyridinedicarboxylate and the three aliphatic diols.

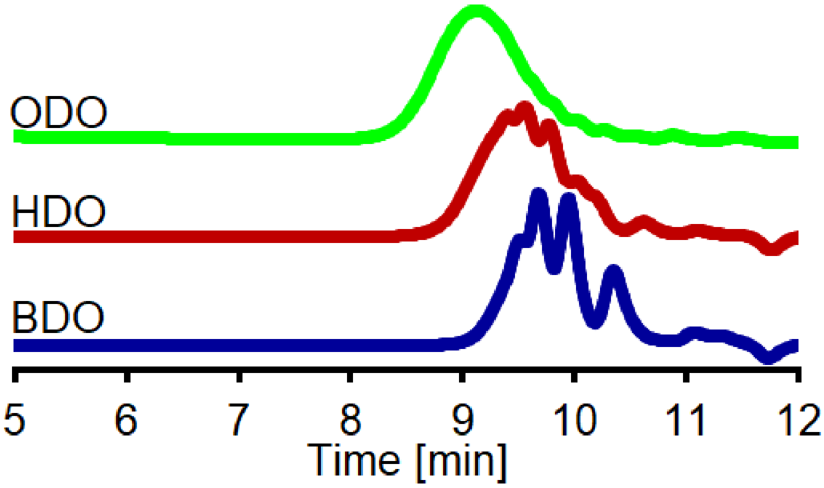

Supplementary Figure 6. GPC chromatograms of the polymers synthesized from diethyl-2,6-pyridinedicarboxylate and the three aliphatic diols.

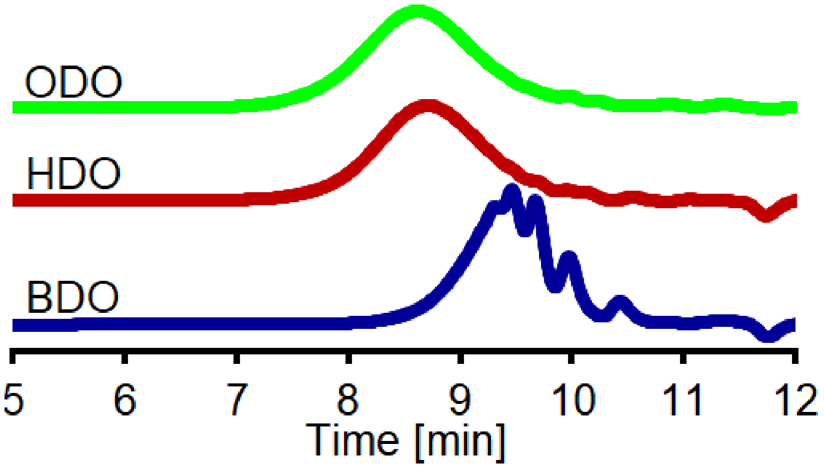

Supplementary Figure 7. GPC chromatograms of the polymers synthesized from diethyl isophthalate and the three aliphatic diols.

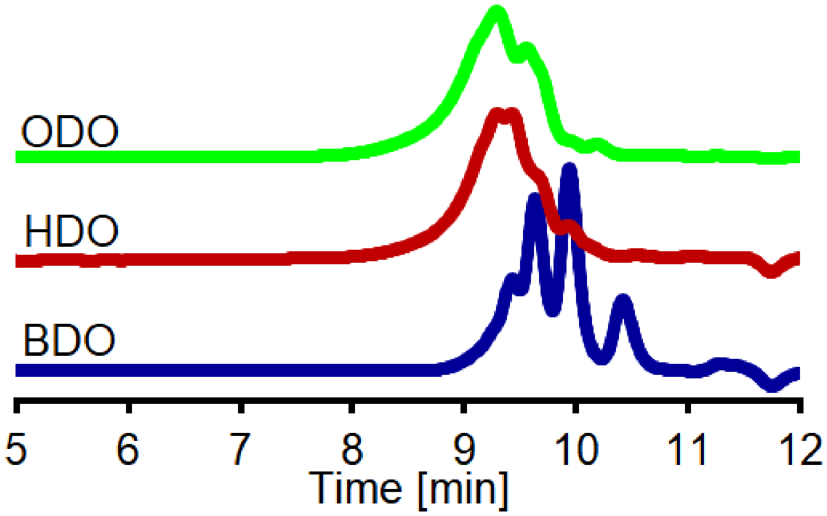

Supplementary Figure 8. GPC chromatograms of the polymers synthesized from diethyl terephthalate and the three aliphatic diols.

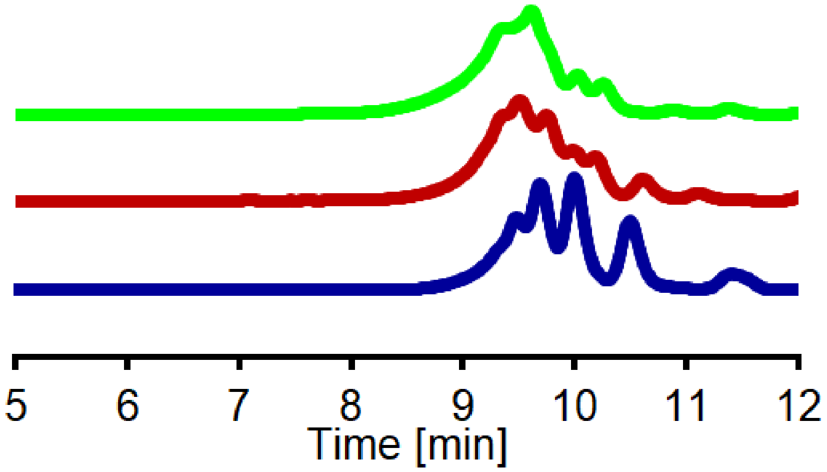

Supplementary Figure 9. GPC chromatograms of the polymers synthesized from diethyl-2.5-furandicarboxylate and the three aliphatic diols.

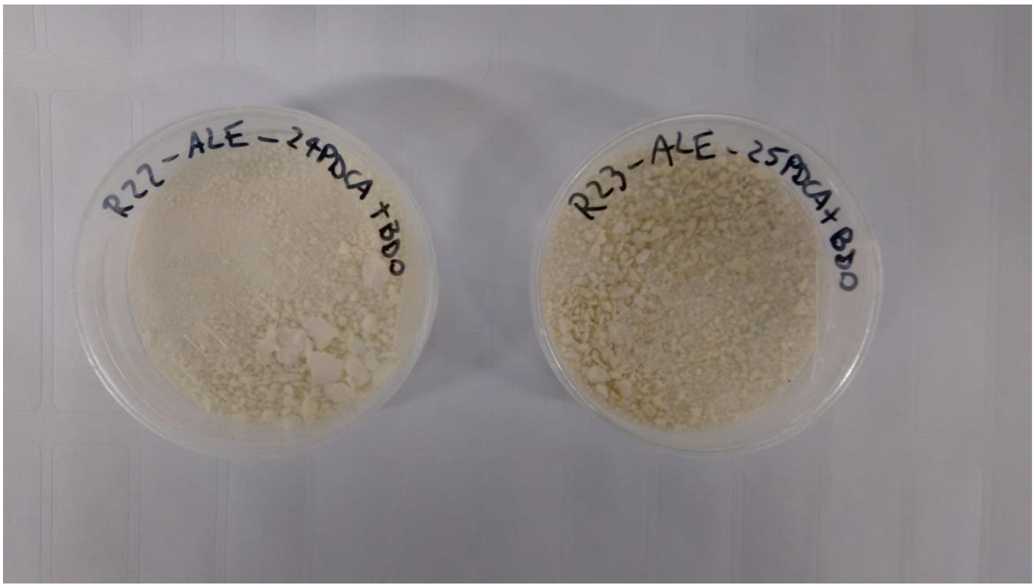

Supplementary Figure 10. Poly(1,4-butylene-2,4-pyridinoate) (left) and poly(1,4-buylene-2,5-pyridinoate) (right) reaction products after CaLB-catalyzed polycondensation reaction in a solventless reaction system.

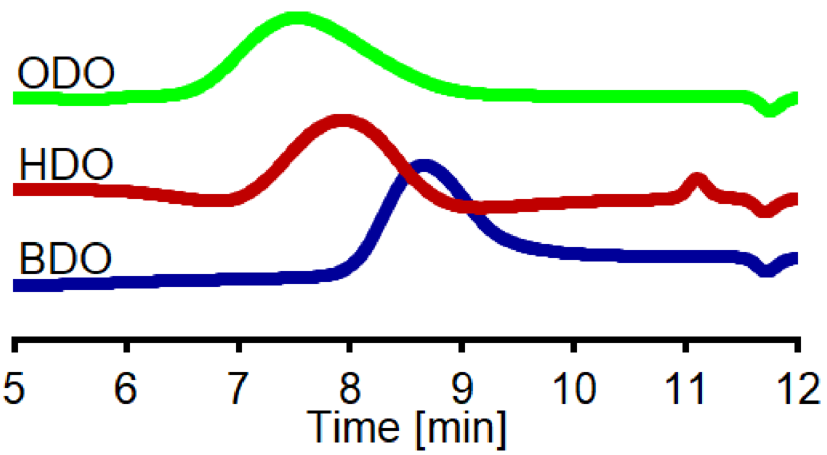

Supplementary Figure 11. GPC chromatograms of the polymers synthesized from diethyl-2,4-pyridinedicarboxylate and the three aliphatic diols in DPE.

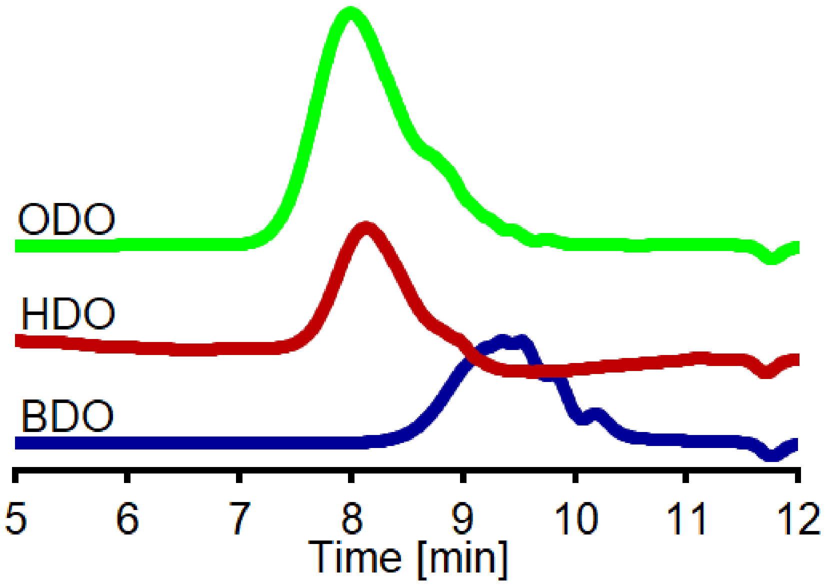

Supplementary Figure 12. GPC chromatograms of the polymers synthesized from diethyl-2,5-pyridinedicarboxylate and the three aliphatic diols in DPE.

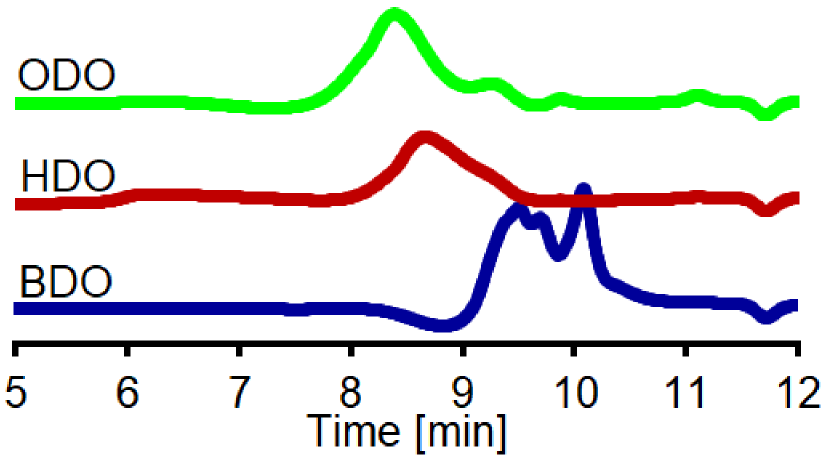

Supplementary Figure 13. GPC chromatograms of the polymers synthesized from diethyl-2,5-pyridinedicarboxylate and the three aliphatic diols in DPE.

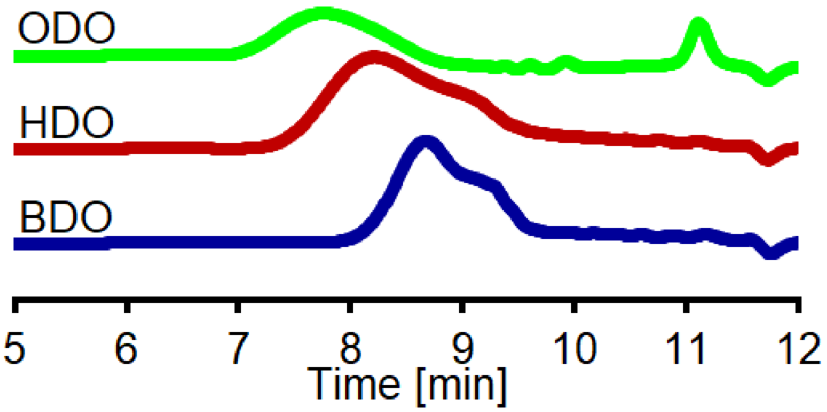

Supplementary Figure 14. GPC chromatograms of the polymers synthesized from diethyl isophthalate and the three aliphatic diols in DPE.

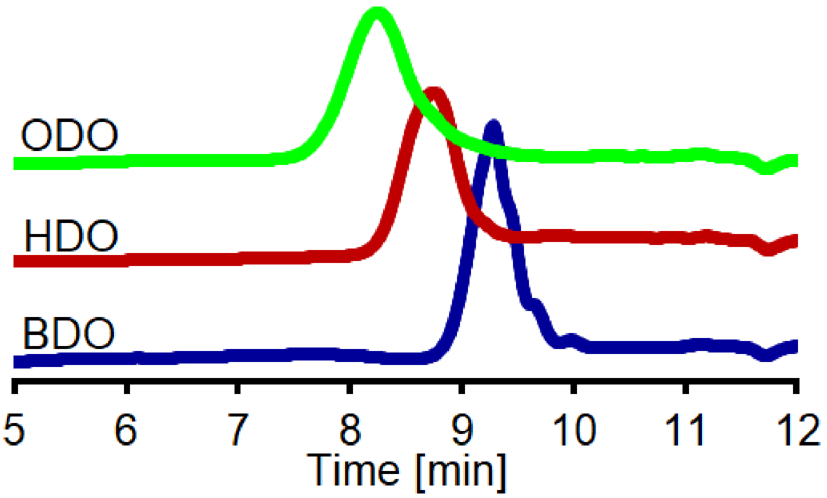

Supplementary Figure 15. GPC chromatograms of the polymers synthesized from diethyl terephthalate and the three aliphatic diols in DPE.

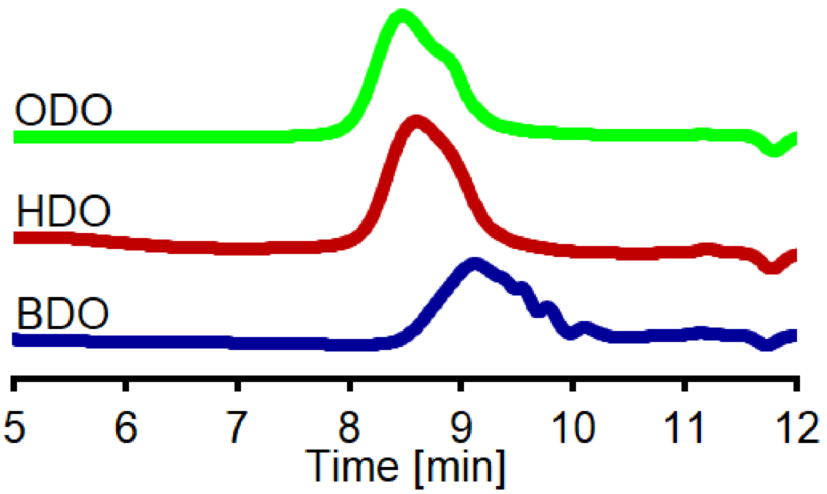

Supplementary Figure 16. GPC chromatograms of the polymers synthesized from diethyl-2,5-furandicarboxylate and the three aliphatic diols in DPE.

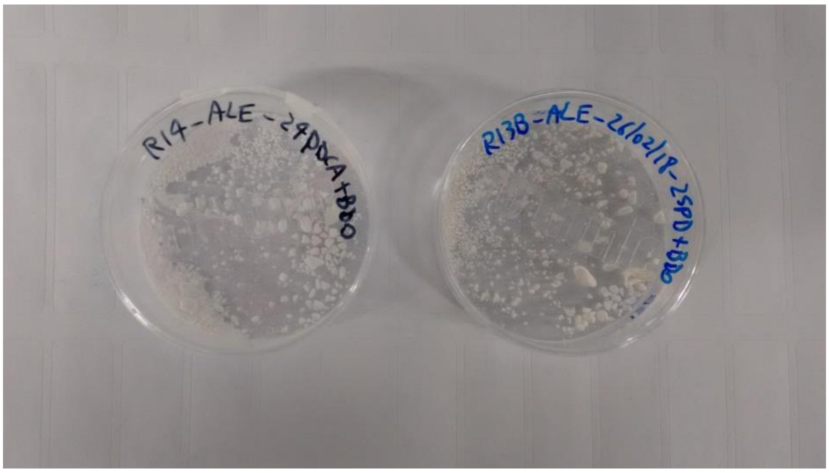

Supplementary Figure 17. Poly(1,4-butylene 2,4-pyridinoate) (left) and poly(1,4-buylene 2,5-pyridinoate) (right) reaction products after CaLB-catalyzed polycondensation reaction in DPE and subsequent MeOH precipitation/purification procedure.

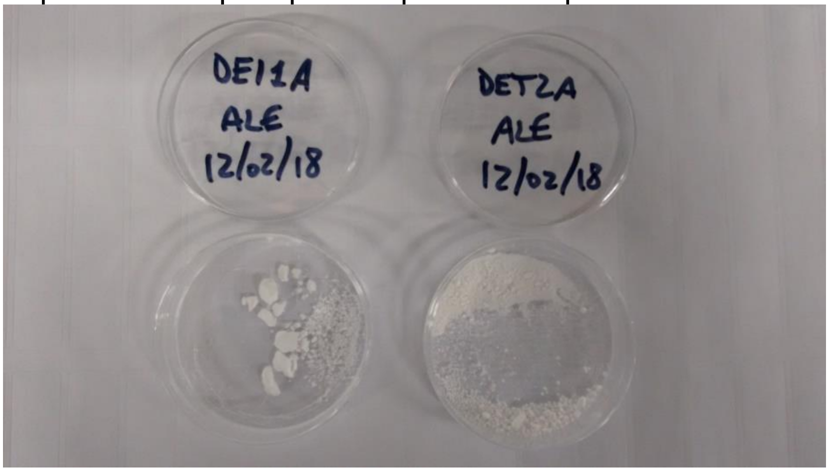

Supplementary Figure 18. Poly(1,4-butylene isophthalate) (left) and poly(1,6-hexylene terephthalate) (right) reaction products after CaLB-catalyzed polycondensation reaction in DPE and subsequent MeOH precipitation/purification procedure.

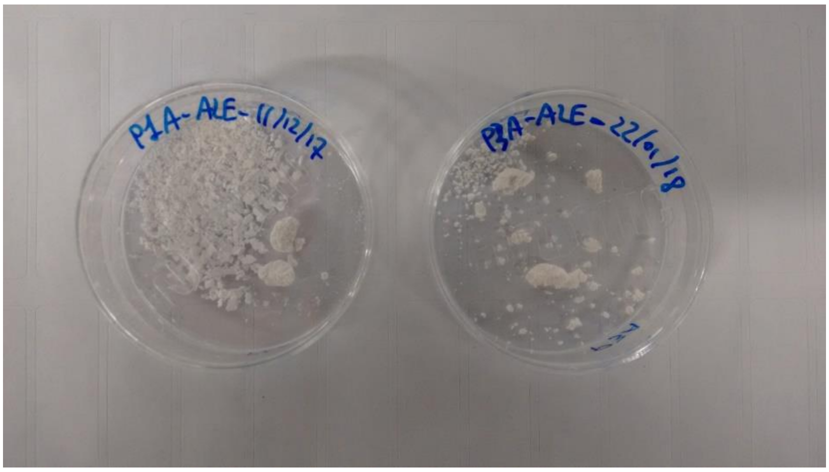

Supplementary Figure 19. Poly(1,6-hexylene 2,4-pyridinoate) (left) and poly(1,8-octylene 2,4-pyridinoate) (right) reaction products after CaLB-catalyzed polycondensation reaction in DPE and subsequent MeOH precipitation/purification procedure.

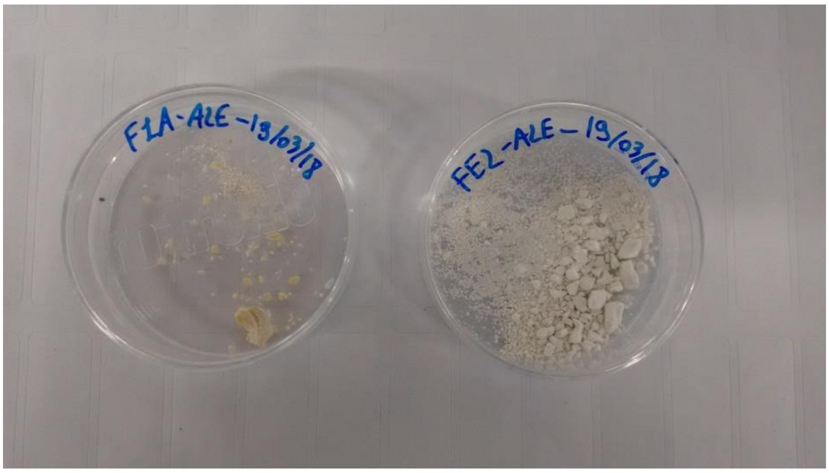

Supplementary Figure 20. Poly(1,4-butylene 2,5-furanoate) (left) and poly(1,6-hexylene 2,5-furanoate) (right) reaction products after CaLB-catalyzed polycondensation reaction in DPE and subsequent MeOH precipitation/purification procedure.

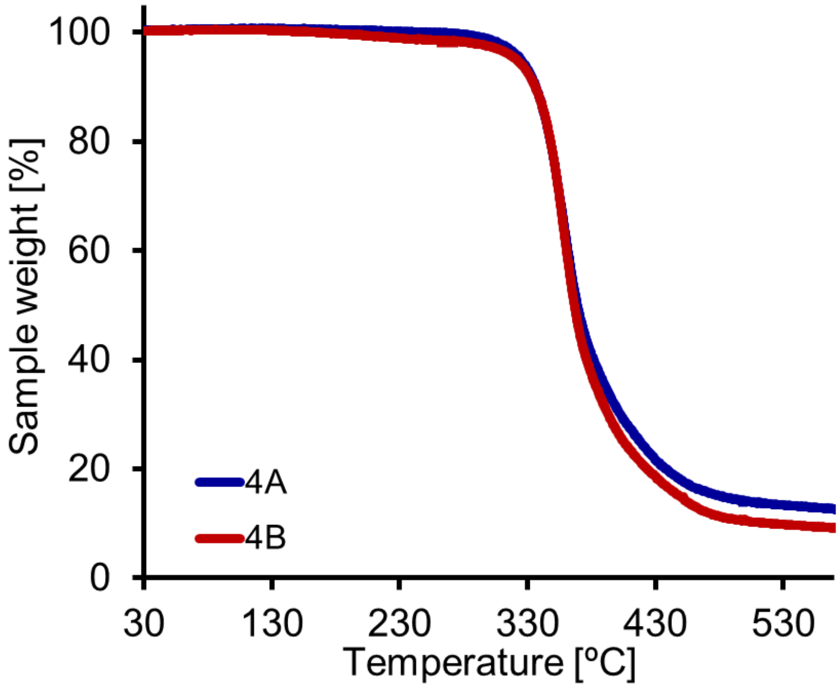

Supplementary Figure 21. TGA analysis of the polymers synthesized from PD24 and 1,4-butanediol in DPE using immobilized CaLB as the biocatalyst.

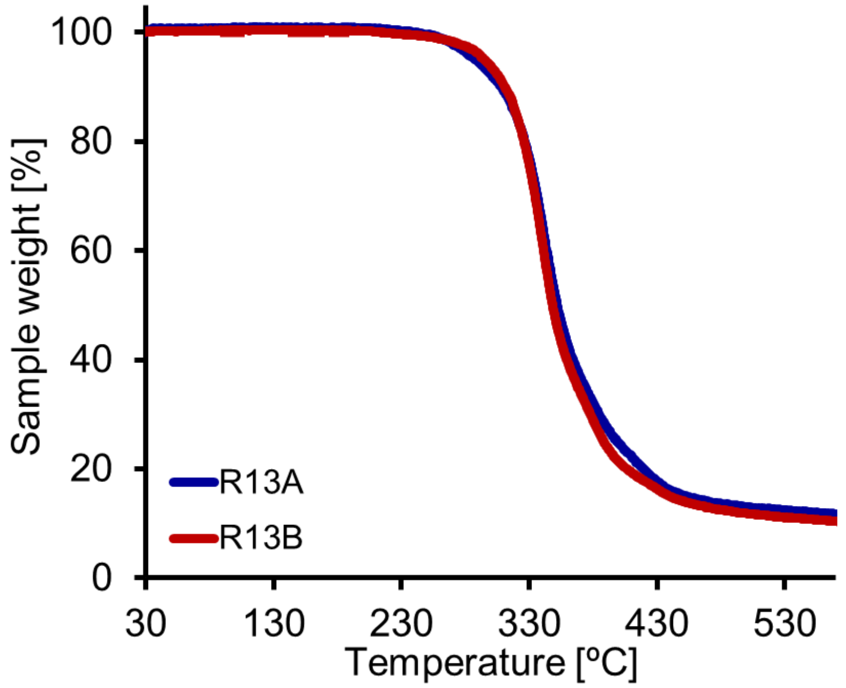

Supplementary Figure 22. TGA analysis of the polymers synthesized from PD25 and 1,4-butanediol in DPE using immobilized CaLB as the biocatalyst.

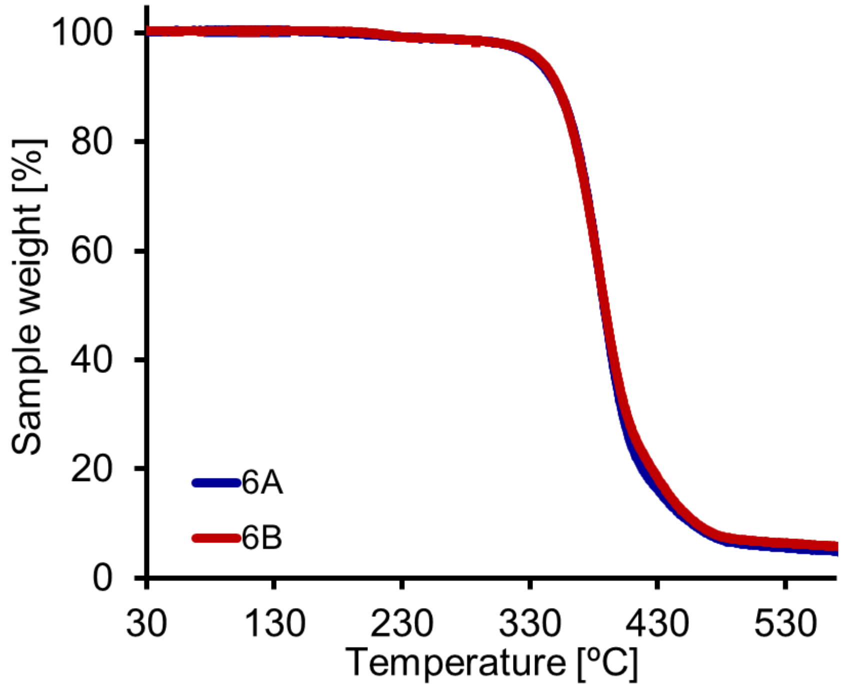

Supplementary Figure 23. TGA analysis of the polymers synthesized from PD26 and 1,8-octanediol in DPE using immobilized CaLB as the biocatalyst.

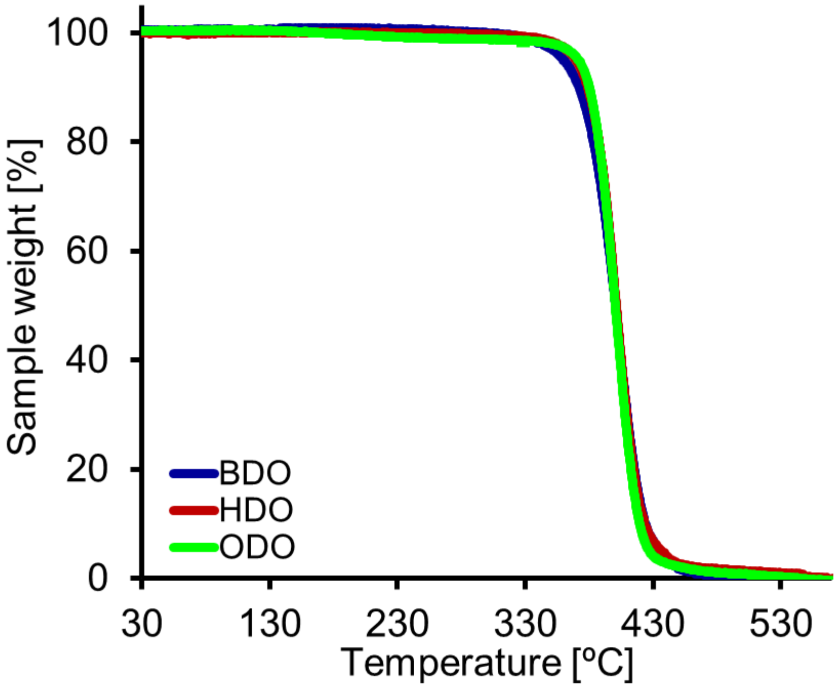

Supplementary Figure 24. TGA analysis of the polymers synthesized from DEI and the three diols (C4, blue; C6, red and C8, green) in DPE using immobilized CaLB as the biocatalyst.

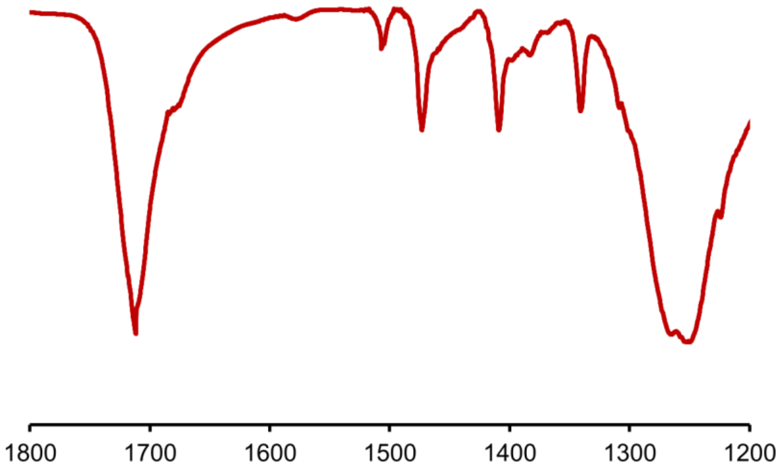

Supplementary Figure 25. FT-IR of the polymer synthesized from DEI and HDO.

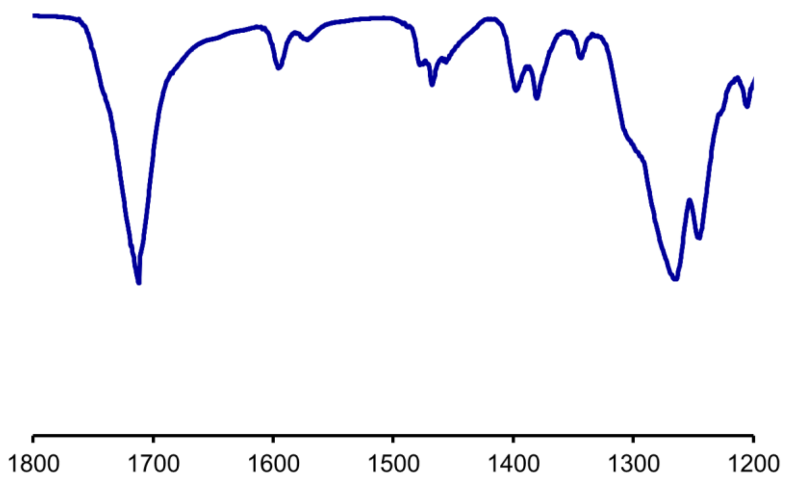

Supplementary Figure 26. FT-IR of the polymer synthesized from PD25 and HDO.

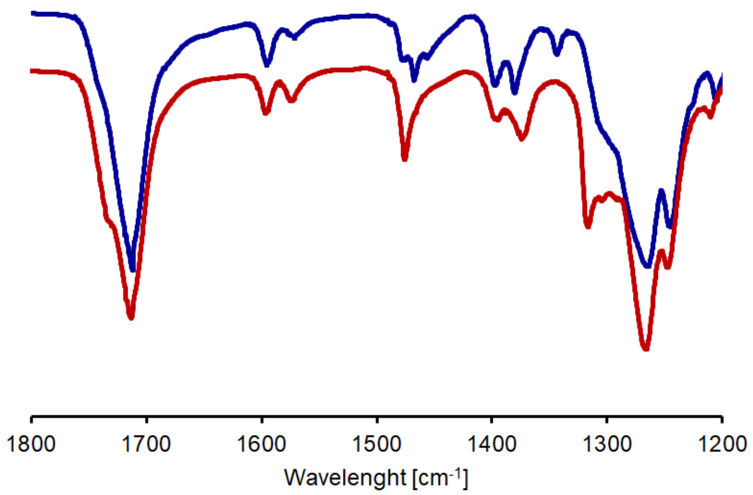

Supplementary Figure 27. Overlayed FT-IR of the polymer synthesized from PD25 and HDO (blue) and ODO (red).

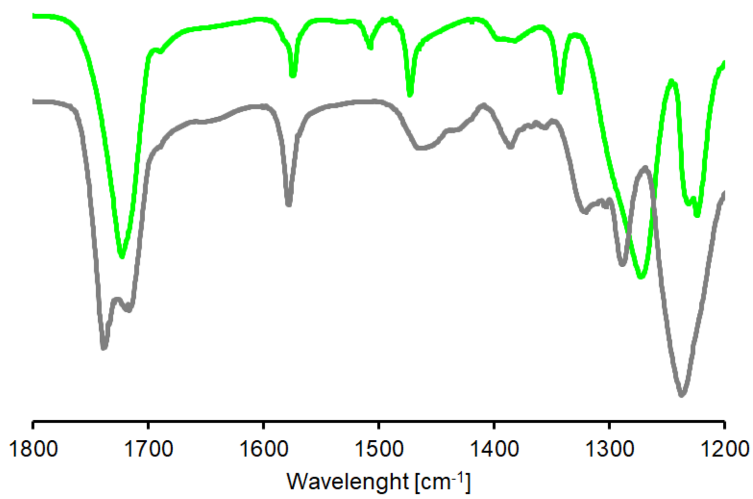

Supplementary Figure 28. Overlayed FT-IR of the polymer synthesized from DEF (green) and PD26 (grey) with HDO as the aliphatic diol.

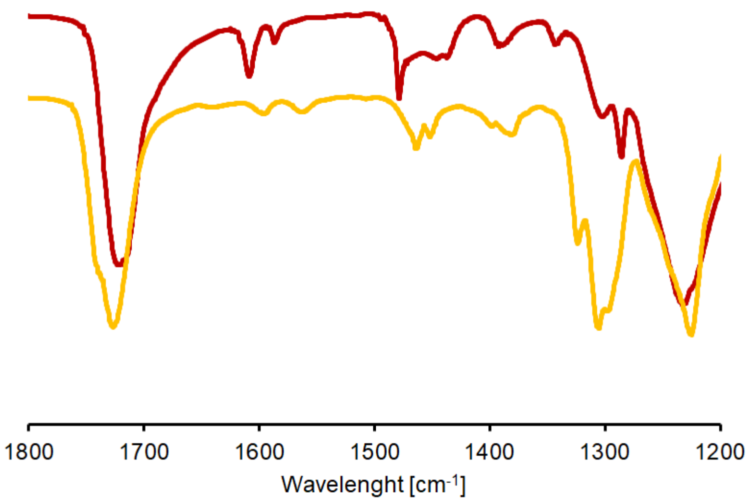

Supplementary Figure 29. Overlayed FT-IR of the polymer synthesized from DEI (red) and PD24 (orange) with HDO as the aliphatic diol.

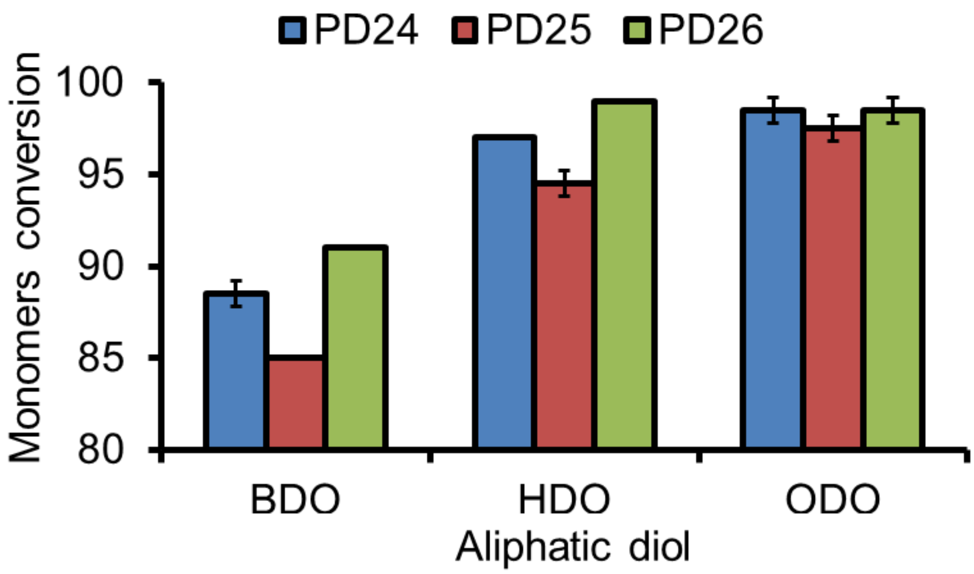

Supplementary Figure 30. Monomers conversion calculated via ^{1}H-NMR (average ± standard deviation) for the reactions between the pyridine diesters and the various aliphatic diols.

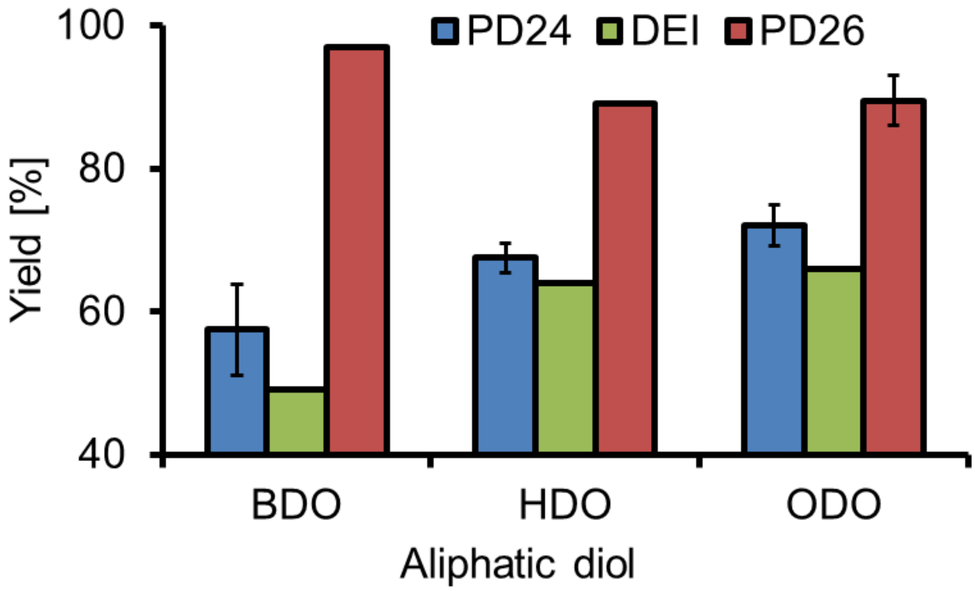

Supplementary Figure 31. Calculated yield and comparison between the PD24, DEI and PD26 aromatic diesters and the various aliphatic diols (average ± standard deviation).

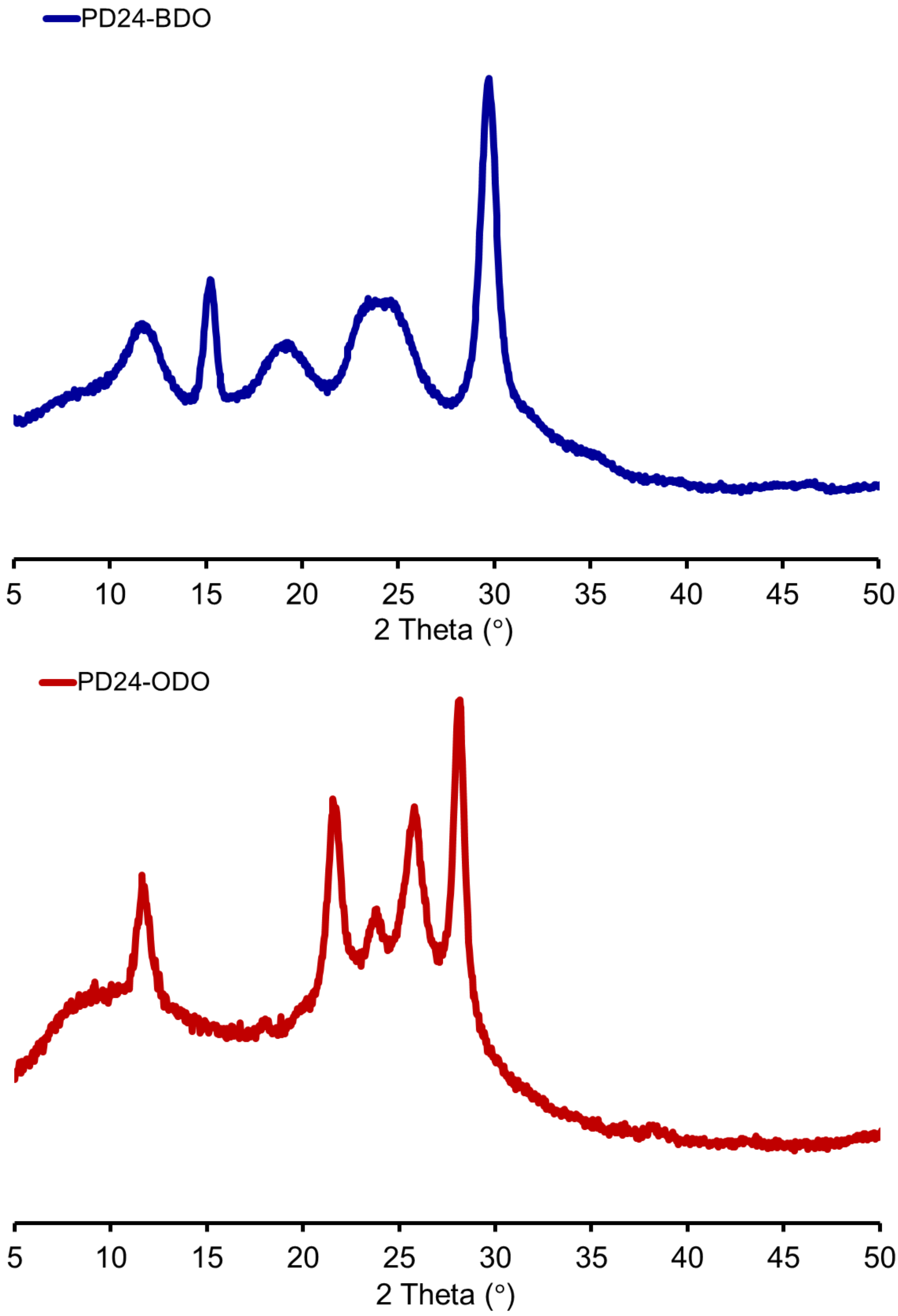

Supplementary Figure 32. XRD analysis of poly(1,4-butylene 2,4-pyridine dicarboxylate) (top) and poly(1,8-octylene 2,4-pyridine dicarboxylate) (bottom).

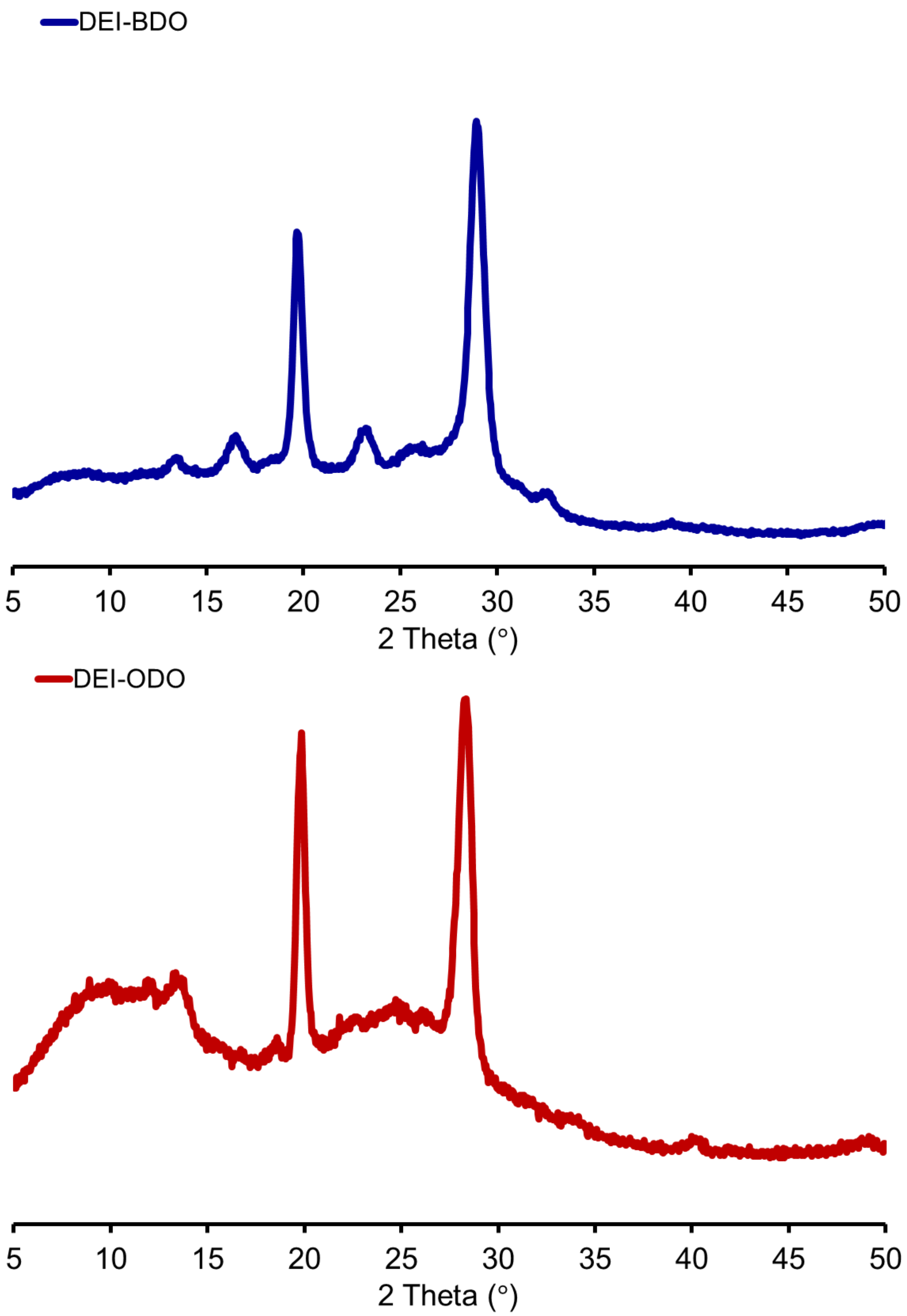

Supplementary Figure 33. XRD analysis of poly(1,4-butylene isophthalate) (top) and poly(1,8-octylene 2,4-pyridine isophthalate) (bottom).

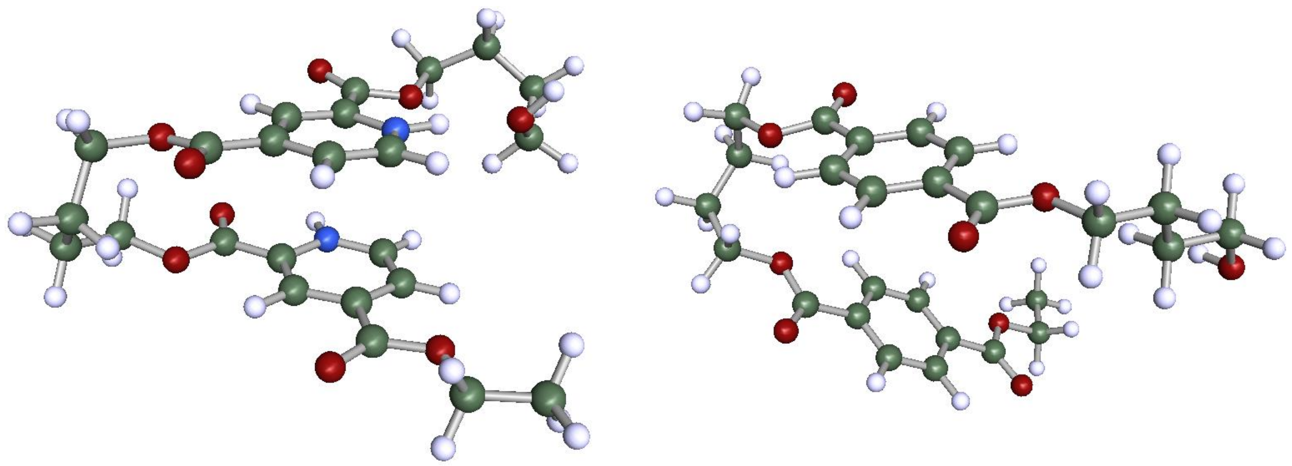

Supplementary Figure 34. COSMO-RS structures of the C_0 conformation of poly(1,4-butylene 2,4-pyridine dicarboxylate) (left) and poly(1,4-butylene isophthalate) (right). Atoms legend: white= hydrogen, green= carbon, red= oxygen, blue= nitrogen.

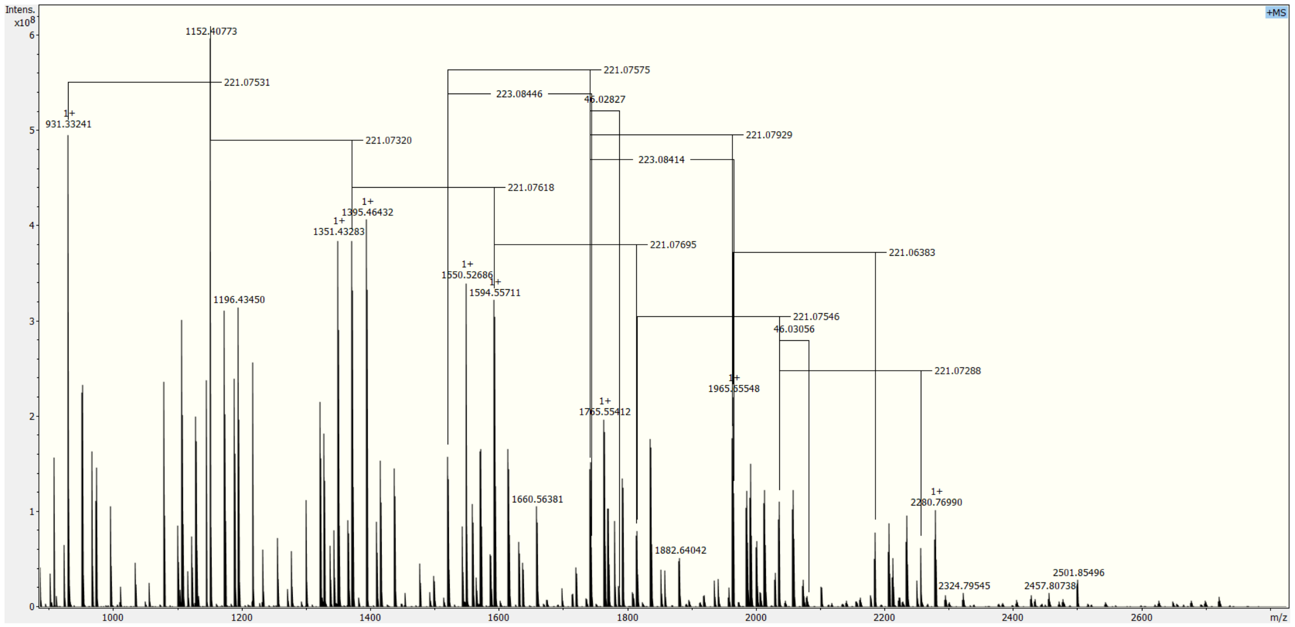

Supplementary Figure 35. MALDI analysis of poly(1,4-butylene 2,6-pyridine dicarboxylate) and assignment of the main peaks.

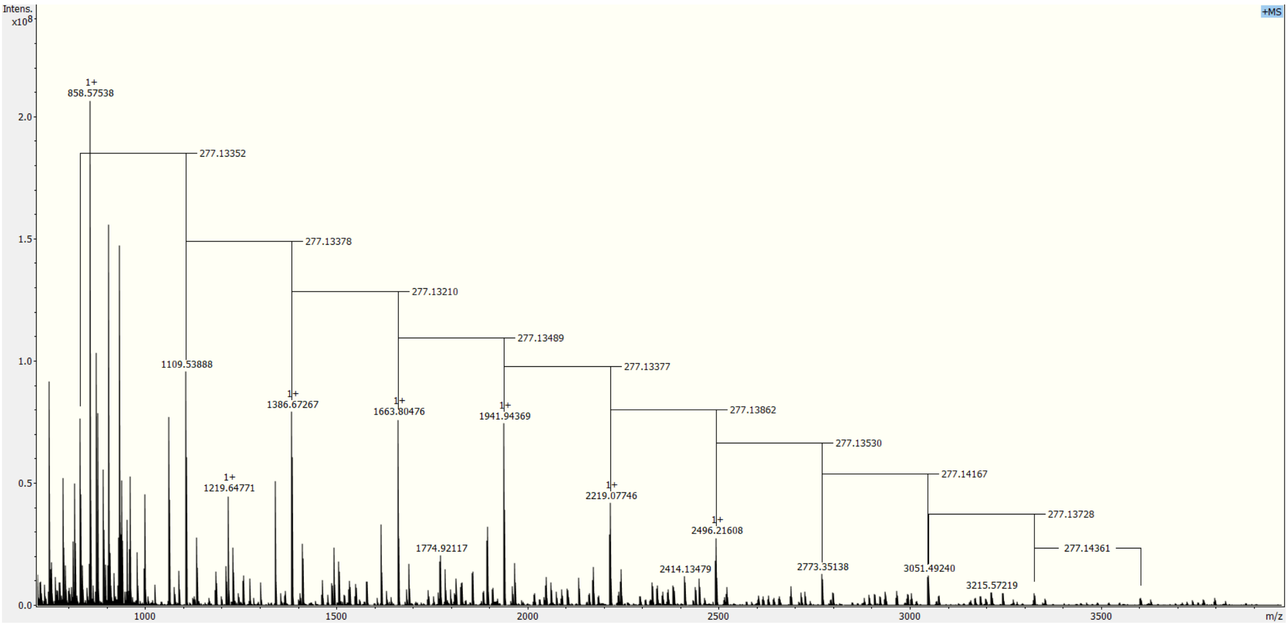

Supplementary Figure 36. MALDI analysis of poly(1,8-octylene 2,4-pyridine dicarboxylate) and assignment of the main peaks.

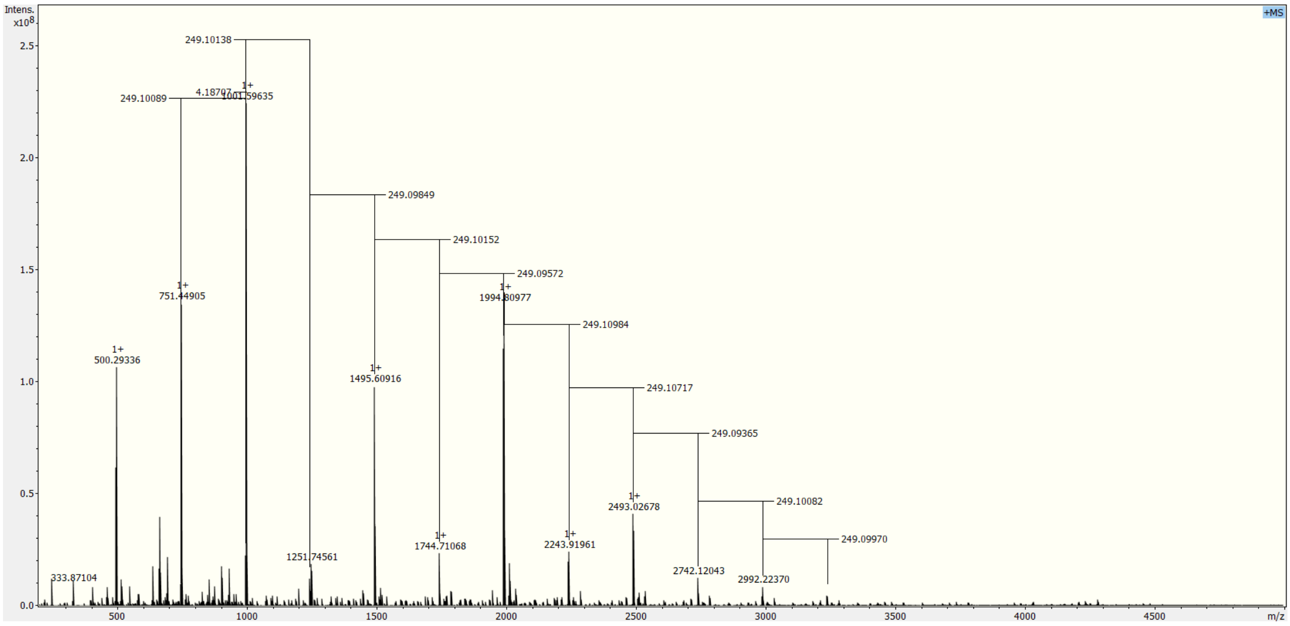

Supplementary Figure 37. MALDI analysis of poly(1,6-hexylene 2,5-pyridine dicarboxylate) and assignment of the main peaks.

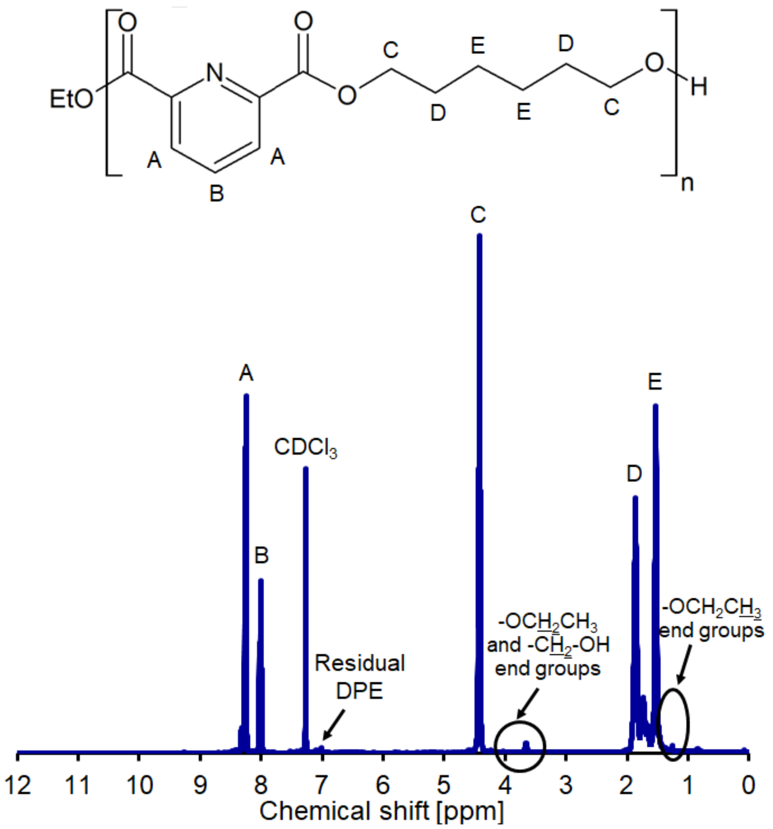

Supplementary Figure 38. Fully assigned ^{1}H-NMR spectra of poly(1,6-hexylene 2,6-pyridinedicarboxylate) synthesized in DPE. All NMR spectra relative to the PD26-based polymers are contained in the associated DOI.

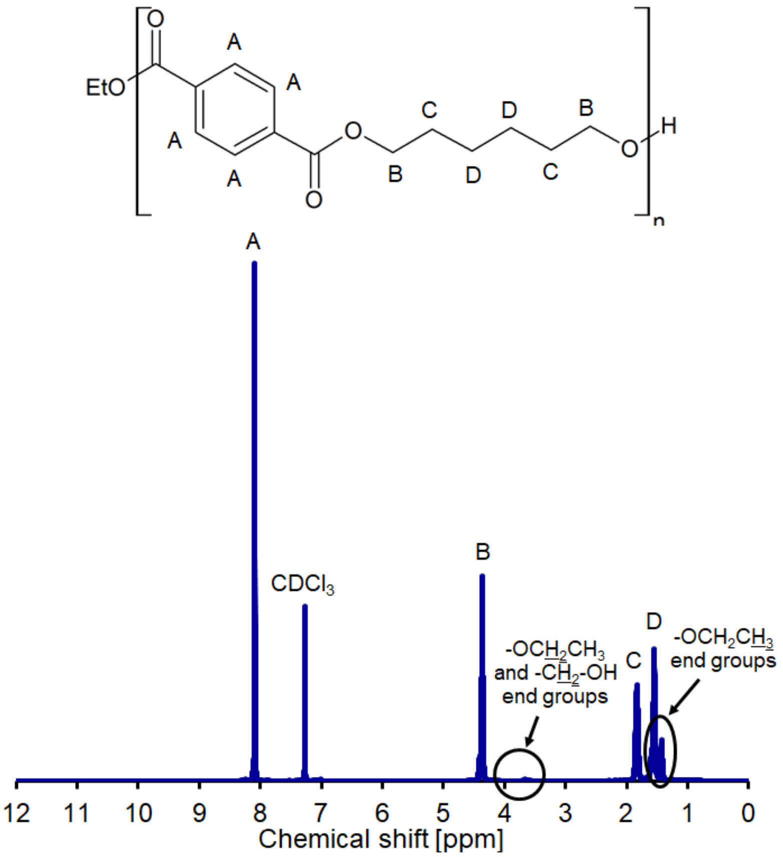

Supplementary Figure 39. Fully assigned ^{1}H-NMR spectra of poly(1,6-hexylene terephthalate) synthesized in DPE. All NMR spectra relative to the DET-based polymers are contained in the associated DOI.

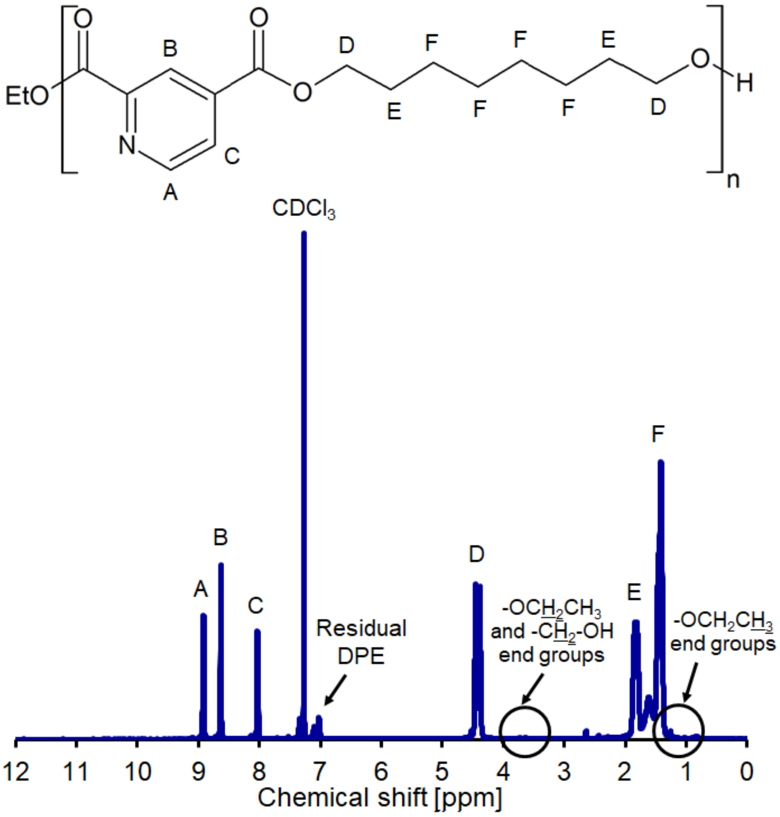

Supplementary Figure 40. Fully assigned ^{1}H-NMR spectra of poly(1,8-octylene 2,4-pyridinedicarboxylate) synthesized in DPE. All NMR spectra relative to the PD24-based polymers are contained in the associated DOI.

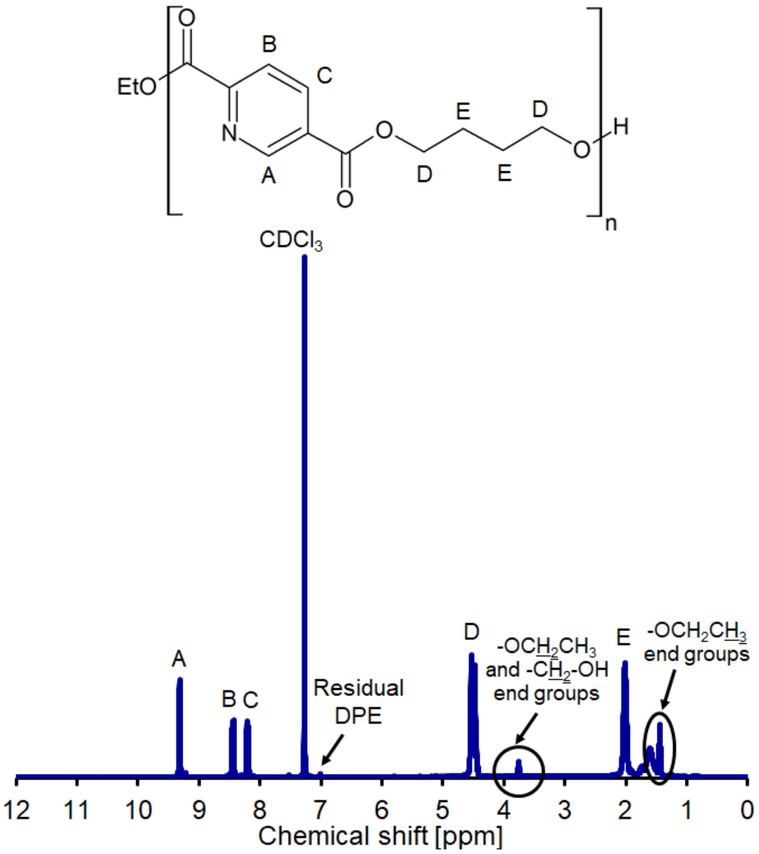

Supplementary Figure 41. Fully assigned ^{1}H-NMR spectra of poly(1,4-butylene 2,5-pyridinedicarboxylate) synthesized in DPE. All NMR spectra relative to the PD25-based polymers are contained in the associated DOI.

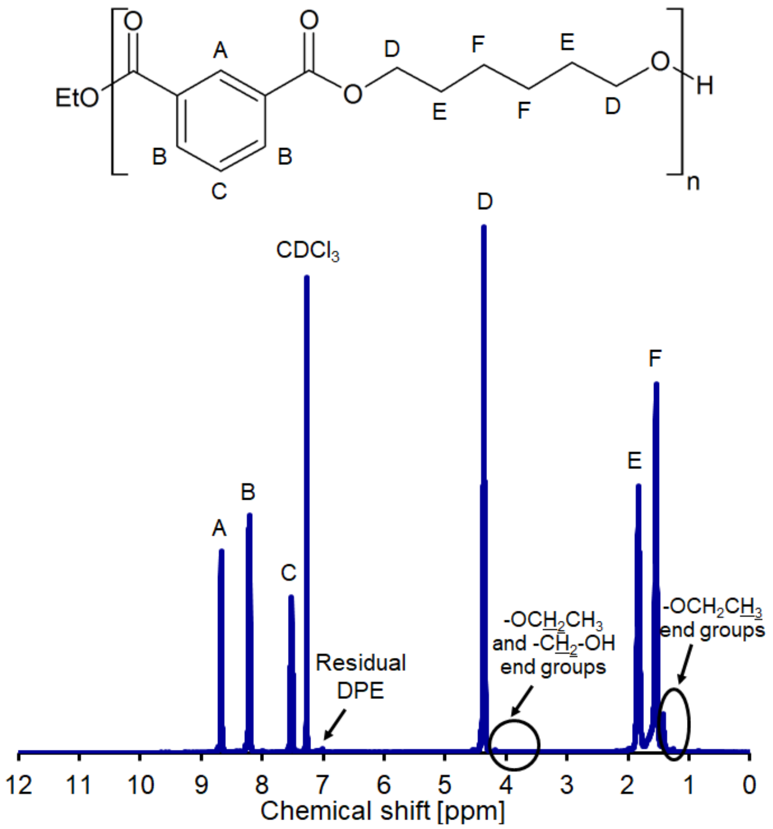

Supplementary Figure 42. Fully assigned ^{1}H-NMR spectra of poly(1,6-hexylene isophthalate) synthesized in DPE. All NMR spectra relative to the DEI-based polymers are contained in the associated DOI.

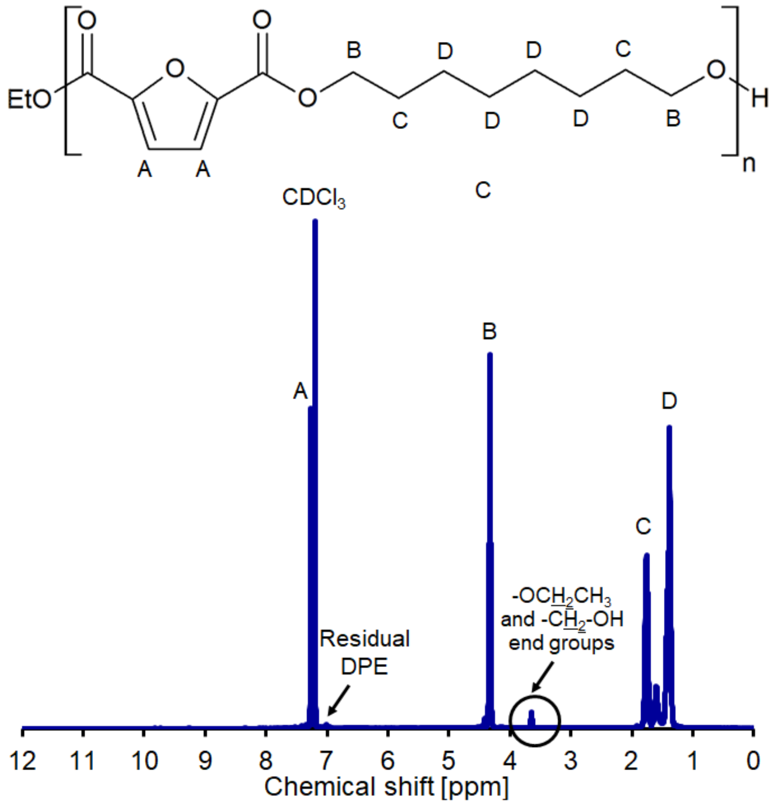

Supplementary Figure 43. Fully assigned ^{1}H-NMR spectra of poly(1,8-octylene 2,5-furandicarboxylate) synthesized in DPE. All NMR spectra relative to the DEF-based polymers are contained in the associated DOI.

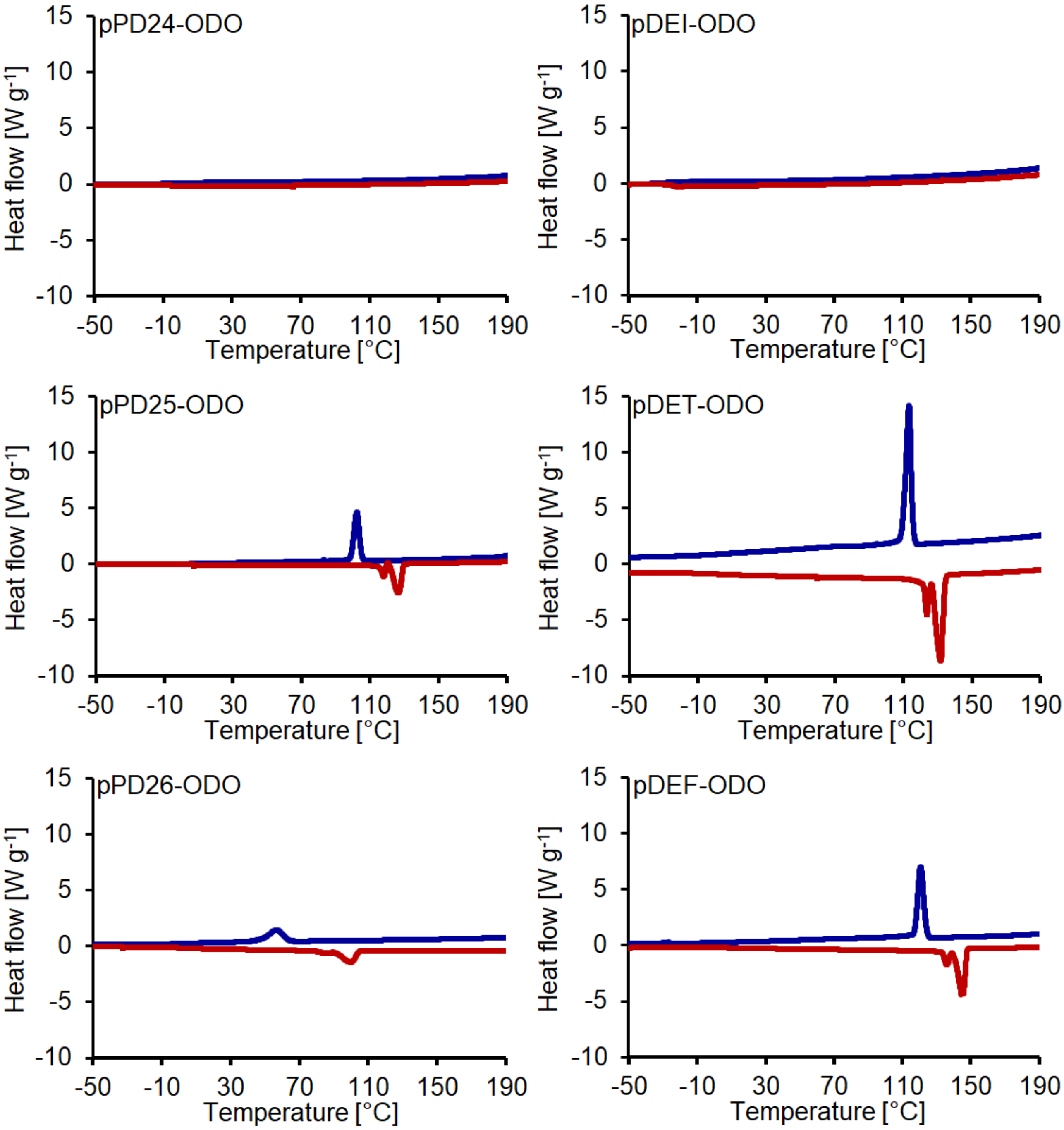

Supplementary Figure 44. DSC thermograms (blue 1st cooling and red 2nd heating cycle) of the polyesters containing ODO as the diol synthesized in the present work.

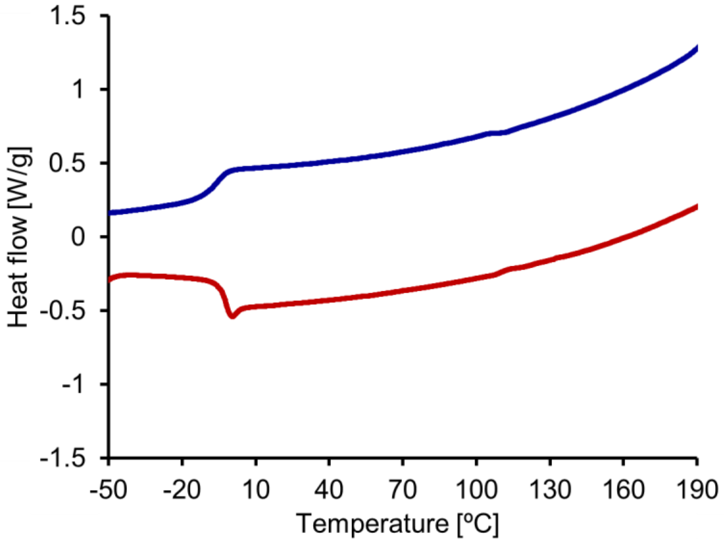

Supplementary Figure 45. Zoom-in of the DSC of poly(1,8-octanediol 2,4-pyridinedicarboxylate). The Y axis shows how weak is the energy of the T_g as also shown in Table S9 where the ΔCp value is reported.

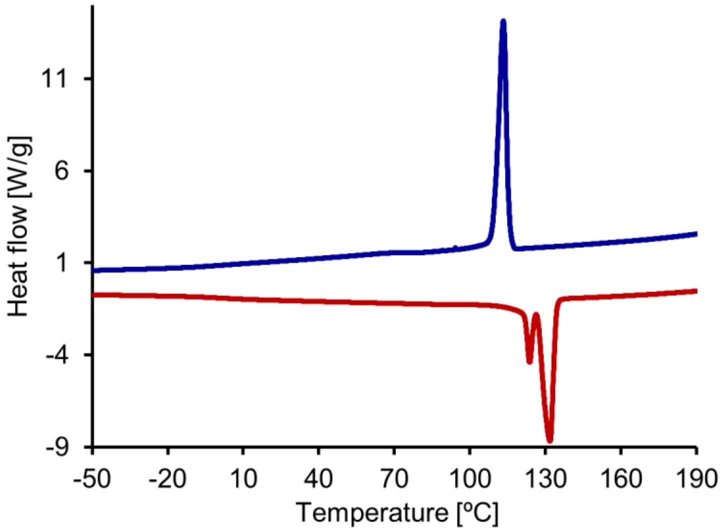

Supplementary Figure 46. DSC of poly(1,8-octanediol terephthalate) as example of DSC of a crystalline polymer.

Supplementary methods

Computational studies

Materials and methods

ArgusLab (version 4.0.1, Mark Thompson and Planaria Software LLC, 2004) was used to draw the 3D models of the oligomers. The relative conformations were then calculated using the COSMOconfX software version 4.0 (COSMOlogic GmbH & Co. KG, 2015). Chemical potentials were generated with COSMOthermX (version C30_1501, COSMOlogic GmbH & Co. KG, 2015). The charge density found on the surface of each molecule was calculated by COSMOthermX to produce a 3D representation, which then determines the corresponding sigma profile and chemical potential plot.

Results and discussion

After experimental data collection, the structures of poly(1,4-butylene 2,4-pyridine dicarboxylate) and poly(1,4-butylene isophthalate) short oligomers (DP2) were selected as model compounds for computational analysis of their chain stacking properties. As shown in Figure 6, while the pyridine rings stack almost perfectly parallel by 180° folding of the polymer chain, the isophthalate-based oligomer stacking is not at all parallel. Furthermore, the proximity of the aromatic rings to one another is closer for the pyridine oligomer compared with the isophthalate equivalent. This is most likely due to intramolecular interactions of the lone pair of the nitrogen; an interaction not possible with a benzene ring. The analysis of the similar DP2 poly(1,4-butylene 2,5-pyridine dicarboxylate) and poly(1,4-butylene terephthalate) gave similar results, with the pyridine 2,5 polymer exhibiting tight stacking with almost perfectly parallel symmetry while the terephthalate-based oligomer stacking was found to be asymmetrical with the rings further apart from each other.
